# Non-invasive prediction of NSCLC immunotherapy efficacy and tumor microenvironment through unsupervised machine learning-driven CT radiomic subtypes: a multi-cohort study

**DOI:** 10.1097/JS9.0000000000002839

**Published:** 2025-06-24

**Authors:** Yusheng Guo, Bingxin Gong, Yi Li, Peng Mo, Yiqun Chen, Qianqian Fan, Qing Sun, Lianwei Miao, Yuanxi Li, Yunting Liu, Wei Tan, Lian Yang, Chuansheng Zheng

**Affiliations:** a Department of Radiology, Union Hospital, Tongji Medical College, Huazhong University of Science and Technology, Wuhan, China; b Hubei Provincial Clinical Research Center for Precision Radiology & Interventional Medicine, Wuhan, China; c Hubei Key Laboratory of Molecular Imaging, Wuhan, China; d Department of Radiotherapy, Fuzong Clinical Medical College (900th Hospital), Fujian Medical University, Fuzhou, China; e Department of Respiratory and Critical Care Medicine, Affiliated Hospital of Nantong University, Medical School of Nantong University, Nantong, China; f School of Medicine, Wuhan College of Arts and Science, Wuhan, China; g Department of Medical Imaging, Geriatric Hospital Affiliated with Wuhan University of Science and Technology, Wuhan, China

**Keywords:** immunotherapy, non-small cell lung cancer, radiomics, tumor immune microenvironment, unsupervised machine learning

## Abstract

**Background:**

Radiomics analyzes quantitative features from medical images to reveal tumor heterogeneity, offering new insights for diagnosis, prognosis, and treatment prediction. This study explored radiomics based biomarkers to predict immunotherapy response and its association with the tumor microenvironment in non-small cell lung cancer (NSCLC) using unsupervised machine learning models derived from CT imaging.

**Materials and methods:**

This study included 1539 NSCLC patients from seven independent cohorts. For 1834 radiomic features extracted from 869 NSCLC patients, K-means unsupervised clustering was applied to identify radiomic subtypes. A random forest model extended subtype classification to external cohorts, model accuracy, sensitivity, and specificity were evaluated. By conducting bulk RNA sequencing (RNA-seq) and single-cell transcriptome sequencing (scRNA-seq) of tumors, the immune microenvironment characteristics of tumors can be obtained to evaluate the association between radiomic subtypes and immunotherapy efficacy, immune scores, and immune cells infiltration.

**Results:**

Unsupervised clustering stratified NSCLC patients into two subtypes (Cluster 1 and Cluster 2). Principal component analysis confirmed significant distinctions between subtypes across all cohorts. Cluster 2 exhibited significantly longer median overall survival (35 vs. 30 months, *P* = 0.006) and progression-free survival (19 vs. 16 months, *P* = 0.020) compared to Cluster 1. Multivariate Cox regression identified radiomic subtype as an independent predictor of overall survival (HR: 0.738, 95% CI 0.583–0.935, *P* = 0.012), validated in two external cohorts. Bulk RNA seq showed elevated interaction signaling and immune scores in Cluster 2 and scRNA-seq demonstrated higher proportions of T cells, B cells, and NK cells in Cluster 2.

**Conclusion:**

This study establishes a radiomic subtype associated with NSCLC immunotherapy efficacy and tumor immune microenvironment. The findings provide a non-invasive tool for personalized treatment, enabling early identification of immunotherapy-responsive patients and optimized therapeutic strategies.

## Introduction

Lung cancer is one of the malignant tumors with the highest incidence and mortality rates globally, with non-small cell lung cancer (NSCLC) representing approximately 85% of all cases^[1,2]^. Although traditional therapies such as surgery, radiotherapy, and conventional chemotherapy have achieved certain therapeutic effects in early and intermediate-stage NSCLC patients^[3]^, the prognosis for traditional treatment plans in advanced NSCLC patients remains dismal^[4]^. In recent years, the advent of immune checkpoint inhibitors (ICIs) has brought significant changes to the treatment of advanced NSCLC^[5]^. ICIs enhance the body’s immune response to tumors by blocking the signaling pathways of immune checkpoint proteins such as programmed cell death-1 (PD-1), programmed death-ligand 1 (PD-L1), and cytotoxic T lymphocyte antigen 4 (CTLA-4), thereby restoring anti-tumor T cell activity to overcome immune evasion^[6]^. This immunotherapy-based approach has shown significant efficacy in some advanced NSCLC patients and can even achieve long-term survival^[7]^. However, immunotherapy efficacy variables widely, with only 20–30% of patients achieving durable responses^[8]^. Furthermore, immune-related adverse events may compromise treatment tolerance and safety^[9]^. Therefore, how to accurately screen patients who may benefit from immunotherapy before treatment and avoid unnecessary treatment-related toxicity is one of the key issues in current clinical practice and research.

Radiomics, as an emerging interdisciplinary field, can reflect the intrinsic heterogeneity of tumors by extracting and analyzing high-dimensional imaging features from medical images, and provide a new perspective for diagnostic classification, prognostic stratification, and therapeutic response prediction^[10]^. The extraction of radiomic features is based on multi-dimensional information such as tumor morphology, texture, and signal intensity, which may be closely related to the biological behavior of tumors, genomic alterations, gene expression profiles, and the tumor immune microenvironment^[11–13]^. Radiomics has achieved notable advancements in oncology research over recent years, especially in predicting tumor invasiveness, recurrence risk, and therapeutic response^[14–16]^. However, although some studies have attempted to apply radiomic to the prediction of immunotherapy efficacy in NSCLC, most remain limited to single-center, small-sample, and lack in-depth exploration of the potential connection between radiomic features and the biological underpinnings, specifically their interplay with the tumor immune microenvironment^[17–19]^. In addition, there are significant differences among different studies in the radiomic feature extraction protocols, analytical workflows, and result interpretation across studies, which hindered clinical translation and broader adoption. Therefore, large-scale, multi-center studies integrating advanced radiomic analysis techniques with biological validation methods are imperative to elucidate the mechanistic links between imaging biomarkers and immunotherapy outcomes in NSCLC.

Based on the above background, this study aims to conduct a multi-center, large-sample cohort study, combining radiomic analysis and single-cell sequencing technology, to investigate the relationship between radiomic features and the efficacy of immunotherapy in NSCLC, and further reveal its potential biological basis. We included patients with advanced NSCLC from multiple medical centers and collected detailed clinical data and chest CT imaging data. Through unsupervised clustering analysis, we divided patients into different radiomic subtypes and evaluated their association with immunotherapy efficacy. In addition, we also analyzed the connection between radiomic subtypes and the tumor immune microenvironment by combining bulk RNA sequencing (RNA-seq) and single-cell RNA sequencing (scRNA-seq), in order to provide new evidence for individualized precision medicine in NSCLC immunotherapy.

## Methods

### Datasets

**Figure 1.**
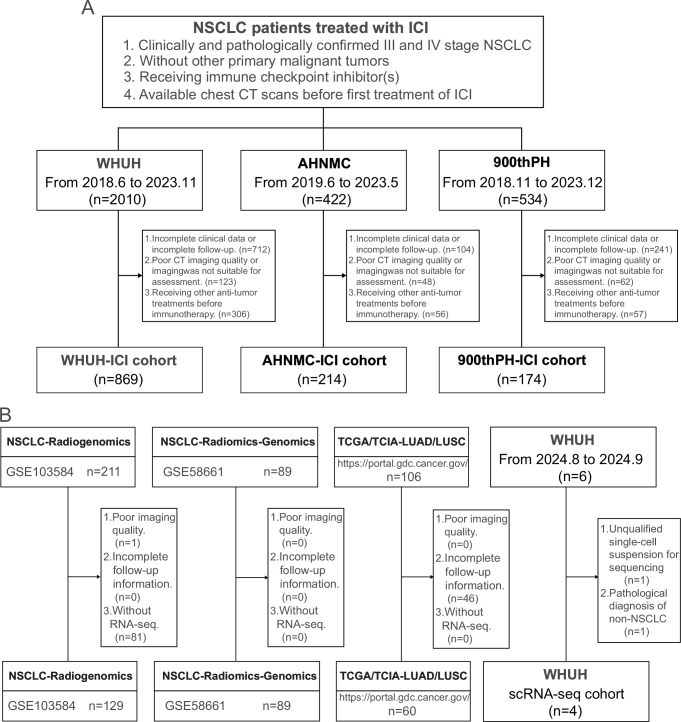
Flowcharts. (A) Flowcharts of patient inclusion and exclusion criteria for A-Hospital-ICI cohort, B-Hospital-ICI cohort, and C-Hospital-ICI cohort. (B) Flowcharts of patient inclusion and exclusion criteria for NSCLC-Radiogenomics cohort, NSCLC-Radiomics-Genomics cohort, TCGA cohort, and A-Hospital (scRNA-seq) cohort.

The study was conducted in accordance with the principles of the Declaration of Helsinki and was approved by the Ethics Committees of the Union Hospital, Tongji Medical College, Huazhong University of Science and Technology (WHUH, A-Hospital), Affiliated Hospital of Nantong University, Medical School of Nantong University (AHNMC, B-Hospital), and the 900th Hospital of the Joint Logistics Team (900thPH, C-Hospital). The inclusion criteria were as follows:^[1]^ Clinically and pathologically confirmed stage III and IV NSCLC,^[2]^ No other primary malignant tumors,^[3]^ Receiving immune check point inhibitors, and^[4]^ Available chest CT scans before the first treatment with ICIs. The exclusion criteria were as follows:^[1]^ incomplete clinical data or incomplete follow-up,^[2]^ poor CT imaging quality or imaging not suitable for assessment, and^[3]^ receiving other anti-tumor treatments before immunotherapy. Additionally, the dataset for analyzing the biological basis consisted of three publicly available databases (NSCLC-Radiogenomics, NSCLC-Radiomics-Genomics, and TCGA) and one prospective cohort initiated by A-Hospital (NCT06500312, Radiogenomics-Lung). In summary, the study included a total of 1539 NSCLC patients from seven independent cohorts. The detailed inclusion and exclusion criteria for each cohort and the patient recruitment process are shown in Fig. 1. The study followed the Transparent Reporting of a Multivariable Prediction Model for Individual Prognosis or Diagnosis (TRIPOD) reporting guidelines^[20]^. This work has been reported in accordance with the updated STROCSS 2025 guideline^[21]^.

**HIGHLIGHTS**

- An unsupervised machine learning model based on CT imaging was developed to predict the response of non-small cell lung cancer to immunotherapy.
- The combination of RNA sequencing and single-cell sequencing revealed the intrinsic connection between radiomics features and the tumor immune microenvironment.
- This study provides a non-invasive and precise stratification tool for clinical practice, which helps to identify patient groups of non-small cell lung cancer that may benefit from immunotherapy.

### Assessment of clinical outcomes and treatment response

Follow-up data were collected from the start of the immunotherapy initiation to the last follow-up across three cohorts. Treatment response was assessed according to the Response Evaluation Criteria in Solid Tumors 1.1^[22]^ at 4–8 weeks and could be classified as complete response (CR), partial response (PR), stable disease (SD), or progressive disease (PD). Progression-free survival (PFS) was defined as the time from the initiation of ICIs treatment to radiologically/clinical confirmed progression or death from any cause. Overall survival (OS) was defined as the time from the start of ICIs treatment to death from any cause. The objective response rate (ORR) was defined as the sum of CR and PR, whereas disease control rate (DCR) was defined as the sum of ORR and SD.

### CT data acquisition, lesion segmentation, and radiomic feature extraction

Chest CT examinations followed a standardized chest scanning protocol that remained consistent across all participating centers and multiple scanner. The information of the scanners at different medical centers is described in Supplemental Digital Content, Table S1 (available at: http://links.lww.com/JS9/E458). All CT images were obtained from the picture archiving and communication system, while the CT images from the three public databases were obtained from the TCIA website, with imaging parameters available on the TCIA website^[23]^. An automatic lung tumor segmentation model trained using nnU-Net^[24]^ was used to segment NSCLC lesions layer by layer on the entire CT scan, obtaining a 3D tumor mask. Subsequently, two intermediate-level radiologists, Q.S. and Q.Q.F. (with 17 and 19 years of experience in pulmonary disease diagnosis, respectively), reviewed and adjusted the segmentation. The former reviewed and adjusted the segmentation for the A-Hospital-ICI cohort and B-Hospital-ICI cohort, while the latter did so for the C-Hospital-ICI cohort, NSCLC-Radiogenomics, NSCLC-Radiomics-Genomics, TCGA, and A-Hospital (scRNA-seq) cohorts. Subsequently, 1834 radiomic features of the tumor were extracted using the “Pyradiomics” Python package, which were divided into five groups:^[1]^ first-order,^[2]^ shape,^[3]^ texture,^[4]^ wavelet, and^[5]^ LoG (Laplacian of Gaussian) features. In cases of multiple tumors, the largest lesion was considered the index tumor.

### Radiomics analysis using unsupervised or supervised learning

The 1834 radiomic features were preprocessed using min-max scaling and de-centered using the ComBat harmonization algorithm which is a harmonization method used to remove scanner and across-centers protocol effects based on the adjusted general linear model harmonization method^[25]^. Subsequently, we performed unsupervised clustering of the radiomic feature matrix for patients in the A-Hospital-ICI cohort using the K-means method (Euclidean distance). This process was carried out using “ConsensusClusterPlus” in R and was repeated 1000 times to ensure the stability of the classification, with the number of clusters determined based on the relative change in the area under the cumulative distribution function curve. Principal component analysis (PCA) was used to verify the components of different radiomic clusters. A random 70% of patients from the A-Hospital-ICI cohort were used as the training set to train a random forest classifier, which is a classifier that performs well in handling high-dimensional data. We implemented the random forest model using the R package “randomForest” (ntree = 100, splitting criteria = “gini”). All 1834 radiomic features were input, and the model was validated in the remaining 30% of the A-Hospital-ICI cohort, with the radiomic clusters extended to other cohorts using this model.

### Biological basis of radiomic clusters

The genes associated with radiomic clusters were identified using the Spearman rank test, with Benjamini–Hochberg multiple testing correction was used to control the false discovery rate (FDR). As previously described^[26]^, we used gene set enrichment analysis (GSEA) to identify Hallmark pathways in three cohorts, which were sorted by FDR, showing the Top 10 pathways in each cohort. Subsequently, we calculated the immune score and microenvironment score for each tumor using “XCell” and estimated the T cell score in the tumor using “MCPcounter” with the three scores compared among the three cohorts. Fresh NSCLC specimens obtained by biopsy or resection were used to construct scRNA-seq libraries using a reverse transcription sequencing protocol, and sequencing was performed on the DNBELAB C4 platform. The R package Seurat was used to analyze scRNA-seq data following published guidelines^[27]^. In summary, gene counts for cells that passed quality control (with fewer than 500 genes detected and mitochondrial percentage under 10%) were normalized by library size and log-transformed using the “NormalizeData” function, followed by the identification of highly variable genes using the “FindVariableFeatures” function, scaling of the expression matrix using the “ScaleData” function, and dimensionality reduction by PCA, with the top 40 informative dimensions selected based on the k-nearest neighbor graph. Uniform manifold approximation and projection (UMAP) were used to visualize the clustering results and further summarize the main principal components. The “Dimplot” function was used to annotate and visualize different cell types, referring to previous literature. We also used the single-sample GSEA (ssGSEA) method to assess the cytotoxic signature and effector signature of cells in T/NK cell subsets, with the detailed genes available in Supplemental Digital Content, Table S2 (available at: http://links.lww.com/JS9/E458), referring to previous studies^[28]^.

### Statistical analysis

Continuous variables were presented as mean ± SD or median (IQR), and categorical variables were expressed as number and percentage. Differences in continuous variables were tested using the Wilcoxon rank-sum test or T-test, and differences in categorical variables were analyzed using the chi-square test or Fisher’s exact test. Survival differences between different groups were evaluated using Kaplan-Meier survival curves and the Log-rank test. Proportional hazards assumptions were performed to determine the independent variables that could be included in the Cox regression model. Subsequently, variables with *P* <0.1 in the univariate model and without multicollinearity (variance inflation factor < 4) were included in the multivariate regression model, and HR and 95% CI were calculated to assess the independent predictors of OS or PFS. Statistical analysis and figure generation were performed using R software (version 4.1.0) and SPSS software (version 26.0), with a two-sided *P* value <0.05 considered statistically significant.

## Result

### Unsupervised machine learning identifies two radiomic subtypes

**Figure 2.**
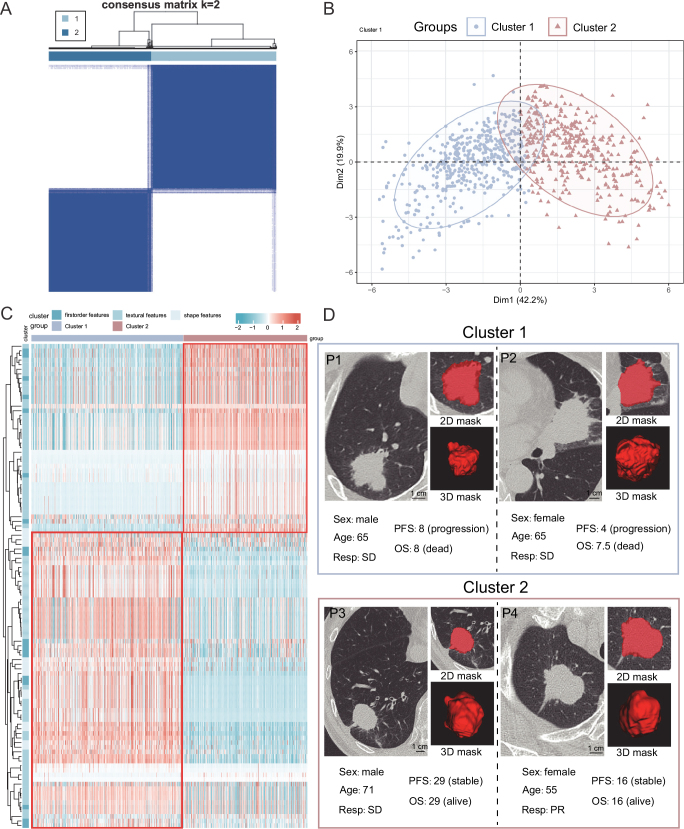
Unsupervised machine learning identifies two radiomic subtypes. (A) Consensus matrix of 869 NSCLC patients when k = 2. (B) The patients from A-Hospital-ICI cohort grouped by two radiomics subtypes (Cluster 1 and Cluster 2) were distinguished by PCA. (C) The heatmap of original radiomic features in A-Hospital-ICI cohort between two radiomics subtypes (Cluster 1 and Cluster 2). (D) Representative CT image with clinical information from different clusters.

K-means clustering of 1834 radiomic features revealed a sharp decline in the relative change of cumulative distribution function area under the curve at *K* = 2 (Supplemental Digital Content, Figure S1, available at: http://links.lww.com/JS9/E458), enabling robust stratification of the A-Hospital-ICI cohort (*n* = 869) into two clusters (Fig. 2A). PCA confirmed distinct radiomic phenotypes (Fig. 2B). When we reclustered using the original radiomic features (*n* = 105), we found a significant difference between Cluster 1 and Cluster 2 (Fig. 2C). In fact, we observed that tumors in cluster 1 were larger, more morphologically variable, and tended to have more spicules, while tumors in cluster 2 were relatively smaller and often round or oval in shape (Fig. 2D). Additionally, we also performed clustering on the A-Hospital-ICI cohort (*n* = 869) using the original 105 features. We found that when using fewer features, the two radiomic subtypes obtained were not able to effectively stratify PFS (Supplemental Digital Content, Figure S2, available at: http://links.lww.com/JS9/E458), which may have been due to the loss of higher-order information. Therefore, Cluster 1 and Cluster 2 derived from the 1835 features were used for subsequent analyses.

### Characteristics of enrolled cohorts

Based on clustering outcomes, NSCLC patients from three medical centers receiving immunotherapy were stratified into Cluster 1 and Cluster 2. In the A-Hospital-ICI cohort (*n* = 869), clusters differed significantly in stage, pathological types, and lower neutrophil-to-lymphocyte ratio (NLR) in Cluster 2 (Supplemental Digital Content, Table S3, available at: http://links.lww.com/JS9/E458). No significant differences between clusters were observed in B-Hospital-ICI cohort and C-Hospital-ICI cohort (Supplemental Digital Content, Tables S4 and S5, available at: http://links.lww.com/JS9/E458).

### Radiomic subtypes predict immunotherapy efficacy and external validation

**Figure 3.**
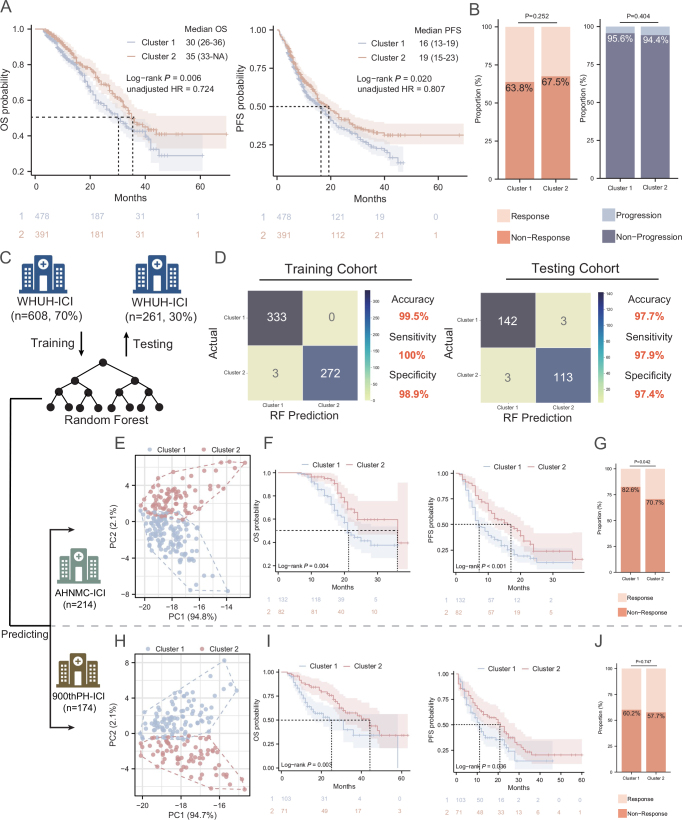
Radiomic subtypes predict immunotherapy efficacy and external validation. (A) KM survival curves showing OS or PFS for the Cluster 1 and Cluster 2 in the A-Hospital-ICI cohort. (B) The ORR and DCR for the Cluster 1 and Cluster 2 in the A-Hospital-ICI cohort. (C) The training of random forest to predict cluster 1 and cluster 2 in the A-Hospital-ICI cohort. (D) The performance of random forest in training cohort and testing cohort. (E) and (H) The patients from B-Hospital-ICI cohort or C-Hospital-ICI cohort grouped by two radiomics subtypes (Cluster 1 and Cluster 2) were distinguished by PCA. (F) and (I) KM survival curves showing OS or PFS for the Cluster 1 and Cluster 2 in the B-Hospital-ICI cohort or C-Hospital-ICI. (G) and (J) The ORR for the Cluster 1 and Cluster 2 in the B-Hospital-ICI cohort or C-Hospital-ICI cohort.

Further investigation of clinical outcomes revealed that Cluster 1 was associated with reduced efficacy of immunotherapy. Through KM survival curves (Fig. 3A), we found that the median survival time was shorter in Cluster 1 (Cluster 1 vs. Cluster 2: 30 months vs 35 months, *P* = 0.006), and the median PFS was also shorter (Cluster 1 vs. Cluster 2: 16 months vs 19 months, *P* = 0.020). Multivariate Cox regression analysis revealed that the radiomic cluster was an independent predictor for OS (0.738 [0.583–0.935], *P* = 0.012; Table 1) and PFS (0.771 [0.640–0.966], *P* = 0.006; Supplemental Digital Content, Table S6, available at: http://links.lww.com/JS9/E458), though no associations emerged with the ORR or DCR (Fig. 3B). To further validate this, we trained a random forest classifier (Fig. 3C) to predict the cluster membership of new samples. With 70% of the patients as the training set (*n* = 608) and 30% of the patients as the testing set (*n* = 261), the overall accuracy of RF reached 99.5% in the training set and 97.7% in the testing set (Fig. 3D). Further analysis of the RF’s feature importance rankings (Supplemental Digital Content, Figure S3, available at: http://links.lww.com/JS9/E458) revealed that the top 10 discriminative features were predominantly advanced-derived parameters, including Gray-level non-uniformity (such as gldm.GrayLevelNonUniformity) and NGTDM texture contrast (such as log.sigma.1.0.mm.3D.ngtdm.Contrast), characterizing intratumoral heterogeneity and tumor-stroma interface complexity. We also evaluated the performance of SVM, XGBoost, and Back Propagation Neural Network (BPNN) classifiers, all of which demonstrated effectiveness comparable to RF, with the AUC values of all four classifiers exceeding 0.9 on the testing set (Supplemental Digital Content, Figure S4, available at: http://links.lww.com/JS9/E458). To ensure methodological consistency, we ultimately selected RF as the classifier for subsequent analyses. In the B-Hospital-ICI cohort, similarly, PCA demonstrated the validity of the classification (Fig. 3E), and we found that OS and PFS were significantly different among different clusters (Fig. 3F, Supplemental Digital Content, Table S7, available at: http://links.lww.com/JS9/E458, and Supplemental Digital Content, Table S9, available at: http://links.lww.com/JS9/E458), with a difference in ORR (Fig. 3G), but not in DCR (Supplemental Digital Content, Figure S5A, available at: http://links.lww.com/JS9/E458). Subsequently, we performed PCA in the C-Hospital-ICI cohort, which also validated the validity of the classification (Fig. 3H), and we confirmed that the radiomic subtypes significantly affected OS and PFS (Fig. 3I, Supplemental Digital Content, Table S8, available at: http://links.lww.com/JS9/E458, and Supplemental Digital Content, Table S10, available at: http://links.lww.com/JS9/E458), but, similar to the A-Hospital-ICI cohort, there was no significant difference in ORR and DCR between radiomic subtypes (Fig. 3J and Supplemental Digital Content, Figure S5B, available at: http://links.lww.com/JS9/E458). Through pooled analysis of data from three centers, we found that radiomic subtypes can significantly stratify patients’ OS across different pathological subtypes of NSCLC (Supplemental Digital Content, Figure S6A, available at: http://links.lww.com/JS9/E458) and clinical stages (Supplemental Digital Content, Figure S6B, available at: http://links.lww.com/JS9/E458), demonstrating the robustness of the radiomic subtyping approach. Moreover, we found that in the subset of patients with PD-L1 information (n = 482) or SUVmax information (n = 226), after adjusting for PD-L1 expression (≤1% or >1% [TPS]), radiomics subtypes remained an independent predictor of OS (adjusted HR = 0.681 [0.491–0.944], *P* = 0.021) and PFS (adjusted HR = 0.698 [0.549–0.887], *P* = 0.003). Following adjustment for SUVmax values (as a continuous variable), radiomics subtypes continued to independently predict OS (adjusted HR = 0.449 [0.285–0.707], *P* < 0.001) and PFS (adjusted HR = 0.598 [0.411–0.87], *P* = 0.007). We also conducted subgroup analyses and found that whether Western or Chinese checkpoint inhibitors were used (Supplemental Digital Content, Figure S7A, available at: http://links.lww.com/JS9/E458), or whether combined with chemotherapy, none of these factors significantly affected the stratification efficacy of radiomics subtypes on OS or PFS (Supplemental Digital Content, Figure S7B, available at: http://links.lww.com/JS9/E458).

**Table 1:** Cox regression analysis of OS in A-Hospital-ICI cohort

Characteristics	Univariate analysis		Multivariate analysis	
	Hazard ratio (95% CI)	*P* value	Hazard ratio (95% CI)	*P* value
**Group**				
Cluster 1	Reference		Reference	
Cluster 2	0.724 (0.575–0.912)	**0.006**	0.738 (0.583–0.935)	**0.012**
**Sex**			
Male	Reference			
Female	1.004 (0.722–1.396)	0.980		
**Stage**				
IV	Reference		Reference	
III	0.749 (0.582–0.965)	**0.025**	0.768 (0.588–1.003)	0.052
**Age**	1.006 (0.993–1.020)	0.375		
**Pathological types**				
Squamous cell carcinoma	Reference			
Adenocarcinoma	1.158 (0.914–1.468)	0.223		
Other	1.101 (0.714–1.696)	0.664		
**Smoking**				
No	Reference			
Yes	1.179 (0.938–1.483)	0.159		
**Drinking**				
No	Reference		Reference	
Yes	1.277 (1.008–1.618)	**0.043**	1.227 (0.959–1.570)	0.103
**BMI**	0.981 (0.944–1.021)	0.351		
**Total bilirubin**	0.998 (0.975–1.022)	0.866		
**LDH**	1.001 (1.001–1.001)	**<0.001**	1.001 (1.000–1.001)	**<0.001**
**NLR**	1.013 (0.999–1.028)	0.077	1.000 (0.981–1.018)	0.978
**PLR**	1.001 (1.000–1.002)	**0.002**	1.001 (1.000–1.002)	**0.026**

BMI: body mass index; LDH: lactate dehydrogenase; NLR: neutrophil-to-lymphocyte ratio; PLR: platelet-to-lymphocyte ratio. Bold font indicates *P* < 0.05.

**Figure 4.**
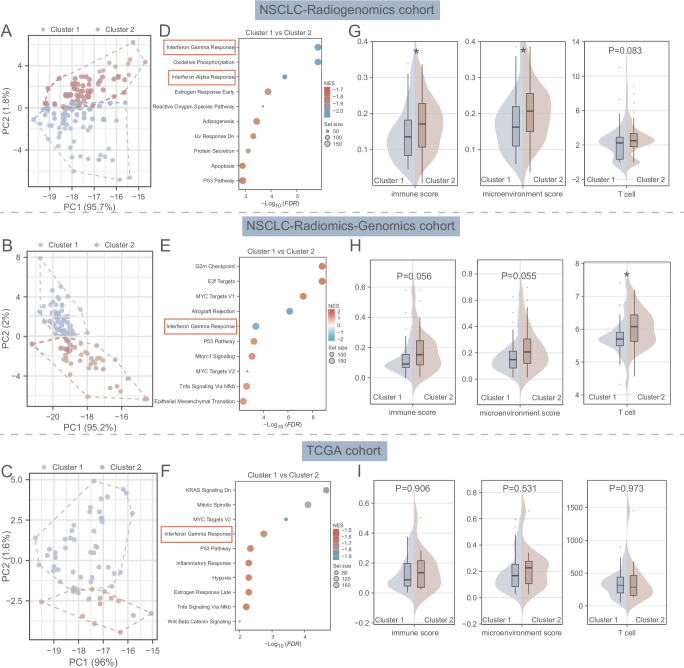
Radiomic subtypes associate with tumor immune microenvironment. (A–C) The patients from NSCLC-Radiogenomics cohort, NSCLC-Radiomics-Genomics cohort, and TCGA cohort grouped by two radiomics subtypes (Cluster 1 and Cluster 2) were distinguished by PCA. (D–F) The top 10 Hallmark pathways related to Radiomics cluster, as revealed by GSEA in the NSCLC-Radiogenomics cohort, NSCLC-Radiomics-Genomics cohort, and TCGA cohort. (G–I) The comparison of the immune score, microenvironment score, and T cell score between cluster 1 and cluster 2 in the NSCLC-Radiogenomics cohort, NSCLC-Radiomics-Genomics cohort, and TCGA cohort.

**Radiomic subtypes associate with tumor immune microenvironment** In three public NSCLC databases, PCA validated radiomic clustering (Fig. 4A**–**C). Cluster 2 consistently exhibited enriched interferon gamma/alpha response pathways (FDR<0.05; Fig. 4D**–**F). Immune and microenvironment scores were significantly elevated in Cluster 2 of the NSCLC-Radiogenomics cohort (*P* = 0.021 and *P* = 0.015; Fig. 4G), with marginally higher T-cell scores (*P* = 0.083). Similar trends were observed in the NSCLC-Radiomics-Genomics cohort (*P* = 0.056 and *P* = 0.055; Fig. 4H), though no differences emerged in TCGA cohort.

### ScRNA-seq uncovers biological basis of radiomics subtypes

**Figure 5.**
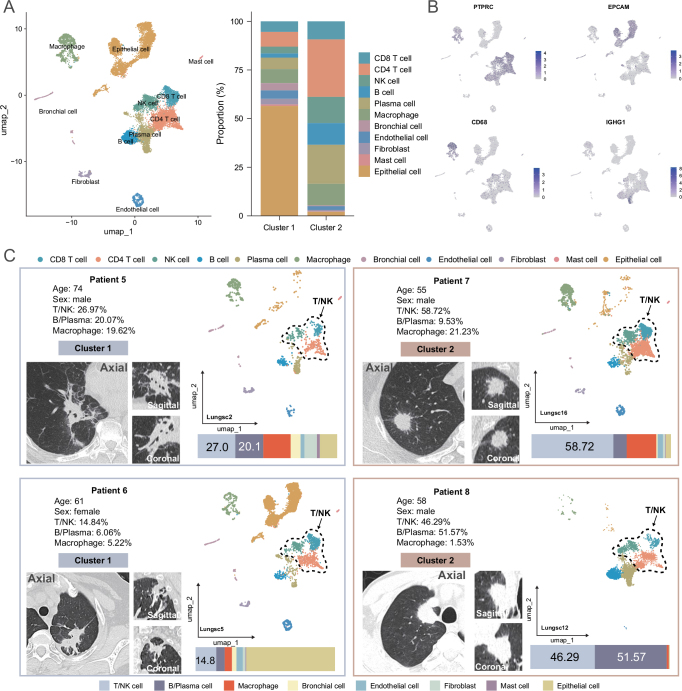
ScRNA-seq uncovers biological basis of radiomic subtypes. (A) UMAP plot of the initial dimensionality reduction, along with cell proportions between different groups. (B) The expression of PTPRC, EPCAM, CD68, and IGHG1. (C) Four representative NSCLC patients CT images, alongside UMAP plot depicting the cellular composition of their tumors. The bar charts display the proportional representation of various cell types.

Single-cell clustering identified major cell populations (Fig. 5A and Supplemental Digital Content, Figure S8A, available at: http://links.lww.com/JS9/E458), including mast cells (*TPSAB1, TPSB2*), bronchial cells (*FOXJ1, SNTN*), fibroblasts (*COL1A1, ACTG2*), endothelial cells (*PECAM1, VWF*), B cells (*CD79A, CD79B*), CD8+ T cells (*CD8A, CD8B*), NK cells (*NKG7, KLRF1*), macrophages (*CD68, C1QA*), plasma cells (*IGHG1, MZB1*), epithelial cells (*EPCAM, KRT7*), and CD4+ T cells (*CD3E, CD4*). It is worth noting that we found a higher proportion of T cells (including CD8+ T cells and CD4+ T cells), NK cells, and B cells in Cluster 2, which may indicate that tumors in different clusters have different immune microenvironments (Supplemental Digital Content, Figure S8B and C, available at: http://links.lww.com/JS9/E458), which may affect tumor behavior and the efficacy of immunotherapy. Figure 5B shows the distribution of the main markers in different cell populations. We analyzed single-cell data from four NSCLC patients (Fig. 5C) and found that in Cluster 1 (P5-6), the proportion of T/NK was relatively low, at 27.0% and 14.8%, respectively, while in Cluster 2 (P7-8), the proportion of T/NK was higher, at 58.7% and 46.3%, respectively. The above results indicate that radiomics clusters are directly related to the immune microenvironment within tumors, and may be primarily based on differences in the infiltration of T cells and NK cells as the main biological basis.

## Discussion

Machine learning has made significant progress in the lung cancer diagnostics and therapeutic strategy development^[29]^. Among them, supervised learning methods, including logistic regression, support vector machines, and other classifiers, remain constrained by limited generalizability, often yielding suboptimal clinical performance. These issues stems from their inherent dependence on tumor subtype-specific training data, which can be greatly affected in real-world clinical settings due to significant differences in tumor immune characteristics or pathological types, limiting their application in broader scenarios^[30]^. To transcend these limitations, this study employed an unsupervised learning algorithms to identify radiomics clusters underlying lung cancer and further explore the relationship between these patterns and patients’ clinical outcomes and immune microenvironment. To our knowledge, this is the first attempt to use CT imaging data to establish a set of radiomics clusters related to the efficacy of NSCLC immunotherapy. Through this approach, we hope to overcome the limitations of existing supervised learning models and provide a new tool for individualized medicine in lung cancer.

This study integrated multi-center radiomics data with transcriptome sequencing, revealing the potential association between CT-based radiomic subtypes and differential immunotherapy outcomes in NSCLC. While the unsupervised clustering model successfully stratified patients into two radiomics subtypes with distinct survival (Cluster 2: median OS 35 months vs. Cluster 1: 30 months, HR = 0.738) and immune infiltration patterns (higher CD8+ T/NK cell densities in Cluster 2). This finding suggests that radiomics features may indirectly predict the long-term efficacy of immunotherapy by reflecting the immune infiltration status of tumors. However, our primary cohort focused on Asian patients, differing from Western radiogenomic datasets. Although the biological rationale (T-cell infiltration as a conserved immunotherapy predictor) may transcend ethnic boundaries^[28,31]^. Therefore, further analysis in Asian patients combining scRNA-seq data found that the enrichment of CD8+ T cells and NK cells in tumors of Cluster 2 patients may be related to their stronger cytotoxic function. These results provide a theoretical basis for the clinical application of radiomics as a non-invasive biomarker, indicating that it cannot only assess tumor heterogeneity at the morphological level but also indirectly reveal the immune biological characteristics of tumors, providing new ideas for individualized treatment decisions.

The findings of this study form an important supplement to the previous applications of radiomics in oncology research^[32]^. Most previous studies primarily linked imaging features to gene mutations or histopathology^[33–35]^, while explorations of immunotherapy efficacy prediction were mostly single-center, small-sample studies lacking in-depth discussion of the underlying biological mechanisms^[17–19]^. To address this issue, this study included data from multi-center immunotherapy cohorts and combined the data from single-cell transcriptomics, demonstrating that CT-based subtypes reflect distinct tumor-immune microenvironment states, especially the differences in infiltration of T cells and NK cells. This finding is consistent with the recent understanding of the mechanisms of immunotherapy response, that is, “hot tumors” with high immune infiltration are more likely to have a sustained response to ICIs^[36,37]^. Previous studies found that imaging semantic features, including tumor size and edge shape, affected the survival of NSCLC and provided explanations from the perspective of gene expression^[38]^. This study further revealed that radiomics features may indirectly reflect the existence of an immune-suppressive microenvironment by capturing the complexity of tumor morphology (such as obvious burrs and border irregularity). For example, tumors in Cluster 1 were larger in volume and had irregular borders (more burrs), which may indicate a higher degree of stromal remodeling and fibrosis^[39,40]^, thereby limiting the infiltration of immune cells (such as T cells, B cells, or NK cells). Consistent with prior studies^[41]^, smooth-bordered lung tumors exhibit reduced expression of epithelial-mesenchymal transition (EMT)-related genes, and this suppression of EMT has been demonstrated to correlate with enhanced immune cell infiltration^[42]^. This hypothesis is consistent with the finding of a higher proportion of fibroblasts in Cluster 1 in scRNA-seq, providing a mechanistic explanation for the association between imaging and biological phenotypes.

Compared with previous studies focusing on specific pathological types or limited clinical scenarios^[43,44]^, a strength of this study is its inclusion of heterogeneous NSCLC populations across pathological types and disease stages (III–IV), furthermore, our multi-center validation demonstrates the robust predictive capacity of radiomic subtypes. This reproducibility underscores the subtypes’ strong generalizability as biomarkers for NSCLC immunotherapy stratification. The radiomics feature analysis in this study was mainly based on non-contrast chest CT, which is a commonly used and relatively cost-effective examination method in clinical practice globally, without any invasive operations. This makes our model more accessible and practical, with the potential to provide effective treatment prediction and guidance for more lung cancer patients even in resource-limited settings. Moreover, radiomic profiling could non-invasively track microenvironment evolution during treatment, complementing static biomarkers like PD-L1 or TMB, and traditional RECIST criteria.

There are noteworthy limitations to this study. First, its retrospective design may introduce selection bias, despite efforts to mitigate heterogeneity through multi-center data integration and ComBat harmonization. Second, the extraction of radiomics features relies on the accuracy of the segmentation algorithm^[45]^. Although the nnU-Net model used in this study was manually verified by radiologists, variations in imaging protocols across institutions could affect reproducibility. Finally, the sample size of scRNA-seq included in this study was limited to a small sample size, limiting the statistical power and generalizability of the results. In the future, we need to verify our conclusions through prospective large-scale clinical trials, assess whether the stratified treatment strategy guided by radiomics can improve patient prognosis, and explore whether radiomics subtypes can add value to existing clinical immunotherapy biomarkers (PD-L1 or TMB). Conducting prospective clinical studies can improve data completeness. In the current study, substantial patient censoring may compromise the robustness and reliability of the analysis, whereas prospective trials enable standardized protocols for follow-up and treatment, thereby reducing data loss and enhancing validity. Moreover, we plan to integrate expanded scRNA-seq, spatial transcriptomics, and multiplex immunofluorescence to dissect molecular mechanisms underlying radiomic subtypes.

## Conclusion

Using unsupervised machine learning algorithms, this study proposed a radiomics subtype closely related to the efficacy of immunotherapy for NSCLC and revealed an association between this subtype and the tumor immune microenvironment. Although retrospective design and limited scRNA-seq data, this study provides a non-invasive assessment tool for personalized treatment of NSCLC, helping to meet the urgent clinical need for early identification of patients who benefit from immunotherapy, thereby optimizing treatment strategies. Prospective validation with intensified monitoring in multi-ethnic cohorts are needed to refine clinical utility and spatial multi-omics is needed to resolve causal radiomics-immune links.

## Data Availability

Data will be available upon reasonable request to the corresponding author.

## Research registration unique identifying number (UIN)

NCT06500312 (https://www.clinicaltrials.gov/).
